# Neuroinflammation induced by lipopolysaccharide causes cognitive impairment in mice

**DOI:** 10.1038/s41598-019-42286-8

**Published:** 2019-04-08

**Authors:** Jiayi Zhao, Wei Bi, Shu Xiao, Xin Lan, Xiaofeng Cheng, Jiawei Zhang, Daxiang Lu, Wei Wei, Yanping Wang, Hongmei Li, Yongmei Fu, Lihong Zhu

**Affiliations:** 1 Department of Pathophysiology, Key Laboratory of State Administration of Traditional Chinese Medicine of the People’s Republic of China, School of Medicine, Jinan University, Guangzhou, Guangdong China; 2 Department of Neurology, The First Affiliated Hospital, Jinan University, Guangzhou, Guangdong China

## Abstract

In this study, we investigated lipopolysaccharide (LPS)-induced cognitive impairment and neuroinflammation in C57BL/6J mice by using behavioral tests, immunofluorescence, enzyme-linked immunosorbent assay (ELISA) and Western blot. We found that LPS treatment leads to sickness behavior and cognitive impairment in mice as shown in the Morris water maze and passive avoidance test, and these effects were accompanied by microglia activation (labeled by ionized calcium binding adaptor molecule-1, IBA-1) and neuronal cell loss (labeled by microtubule-associated protein 2, MAP-2) in the hippocampus. The levels of interleukin-4 (IL-4) and interleukin-10 (IL-10) in the serum and brain homogenates were reduced by the LPS treatment, while the levels of tumor necrosis factor-α (TNF-α), interleukin-1β (IL-1β), prostaglandin E2 (PGE_2_) and nitric oxide (NO) were increased. In addition, LPS promoted the expression of cyclooxygenase-2 (COX-2) and inducible nitric oxide synthase (iNOS) in the brain homogenates. The Western blot analysis showed that the nuclear factor kappa B (NF-κB) signaling pathway was activated in the LPS groups. Furthermore, VIPER, which is a TLR-4-specific inhibitory peptide, prevented the LPS-induced neuroinflammation and cognitive impairment. These data suggest that LPS induced cognitive impairment and neuroinflammation via microglia activation by activating the NF-kB signaling pathway; furthermore, we compared the time points, doses, methods and outcomes of LPS administration between intraperitoneal and intracerebroventricular injections of LPS in LPS-induced neuroinflammation and cognitive impairment, and these data may provide additional insight for researchers performing neuroinflammation research.

## Introduction

Neuroinflammation is an important factor contributing to cognitive impairment and neurodegenerative diseases, including Alzheimer’s disease (AD), Parkinson’s disease (PD), Huntington’s disease, multiple sclerosis (MS) and amyotrophic lateral sclerosis^1,2^.

Microglia, which are the resident macrophages in the brain, have been discovered to play an important role in the occurrence and development of neuroinflammation^3^. Although acute neuroinflammation plays a protective role in the body^4–6^, chronic neuroinflammation is always considered detrimental and damaging to nervous tissue^7,8^. Thus, whether neuroinflammation leads to beneficial or harmful outcomes in the brain may critically depend on both the duration of the inflammatory response and the type of microglia activation^6,9^. Under physiological conditions, microglia mainly eliminate metabolic products and toxic materials. However, if stimulated, microglia migrate to the lesion and remove cellular debris. While microglia activation is necessary and critical for host defense, excessive or prolonged activation of microglia leads to neuronal death and an increase in proinflammatory cytokines, especially in the hippocampus. To date, numerous reports have indicated that Aβ activates microglia and induces their release of proinflammatory cytokines, such as nitric oxide (NO), tumor necrosis factor (TNF)-α and interleukin (IL)-1β, which are hallmarks of AD, PD, MS, and cerebral ischemia^10–16^.

Lipopolysaccharide (LPS; a cell-wall immunostimulatory component of gram-negative bacteria) was first identified as a Toll-like receptor 4 (TLR-4) ligand^17^. TLR-4 is primarily expressed on microglia^18^ in the central nervous system, which once activated, produce proinflammatory cytokines, such as TNF-α, IL-1β, prostaglandin E_2_ (PGE_2_) and NO^19,20^. These cytokines are key mediators of the neuro-inflammatory process. More importantly, TLR-4 mediates extensive neuronal cell death. Incidentally, the TLR-4-specific viral inhibitory peptide VIPER has been shown to potentially inhibit TLR-4-mediated responses induced by LPS^21^. Furthermore, the administration of LPS to animals induces cognitive impairment^22,23^ and a complex array of behaviors, including anorexia, decreased locomotion, weight loss, exploratory behavior, increased anxiety, somnolence, and general behavioral depression. Several of these symptoms are thought to be very similar to the clinically relevant symptoms of neurodegenerative disease in humans. Therefore, the administration of LPS is frequently used to study neuroinflammation-associated diseases in mice. In addition, studies focusing on LPS-induced cognitive impairment often vary in the dose and time of the LPS treatment. However, many studies only use a single injection method to deliver LPS, a few time points and/or a single dose of LPS; thus, assessing different injection methods and time- and dose-dependent changes in neuroinflammation and behaviors is impossible. Furthermore, the underlying mechanisms involved in LPS-induced cognitive impairment in mice are unclear. Here, we induced neuroinflammation via intraperitoneal (i.p.) or intracerebroventricular (i.c.v.) injections of LPS and investigated the possible mechanisms of LPS-induced cognitive impairment by assessing the interaction between Aβ and neuroinflammation^23–25^.

## Materials and Methods

### Animals and treatments

**Figure 1:**
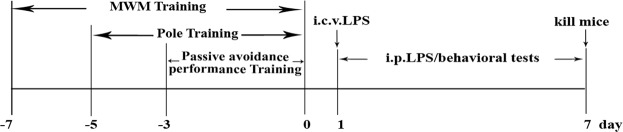
Illustration of the protocols, including the time line of the experiments and tests.

C57BL/6J male mice (11–12 weeks old) were purchased from the Guangdong Medical Laboratory Animal Center. All animal experiments were approved and carried out according to the Animal Ethics Committee of Jinan University (CBNUA-436–12–02). All mice were housed in a room with automatically controlled temperature (21–25 °C), relative humidity (45–65%), and light-dark (12–12 h) cycles. The mice in each cage were divided into the following treatment groups for each of the five experiments: (I) i.p. saline group (control), (II) i.p. LPS (500 μg/kg) group, (III) i.p. LPS (750 μg/kg) group, (IV) i.c.v. saline group, and (V) i.c.v. LPS (12 μg) group. Each group consisted of ten male mice. The i.p. LPS injections were administered at doses of 500 or 750 μg/kg in saline for seven consecutive days, and the saline (0.9% NaCl) control mice received saline injections each day of testing. The i.c.v. injections of 12 μg of LPS (in 3 μL of saline) and saline (control) injections were administered on one day using a microsyringe and stereotaxic coordinates (−2.6 mm dorsal/ventral, −1.5 mm lateral, and −0.2 mm anterior/posterior from bregma) according to the procedure originally described by Haley and McCormick^26^. The mice in the VIPER + LPS group received VIPER (100 μg/kg i.p.) 2 h prior to the LPS injection. After training, testing was performed daily (day 0 to day 7) (Fig. 1).

### Morris water maze test

The Morris water maze (MWM) test, which is a commonly accepted method of testing memory, was performed as described by Morris *et al*.^27^. The MWM program and equipment were purchased from ChengDu Technology & Market Co, LTD. A circular pool (height: 35 cm, diameter: 120 cm) was filled with water rendered opaque with whole milk and maintained at 23 ± 2 °C. An escape platform (height: 14 cm, diameter: 4–5 cm) was submerged 1–1.5 cm below the surface of the water in a specific position. During the training trials, the mice were placed into the water in a random quadrant and allowed to locate the hidden platform for 60 s. Then, the mice were allowed to remain on the platform or were placed on the platform for 10 s at the end of each trial. The mice were trained with three trials per day for 7 days. After the training, on the final day of testing, we administered LPS or saline 6 h before conducting the spatial probe test. The escape latency and escape distance displayed by each mouse were recorded by a computer.

### Passive avoidance performance test

The passive avoidance test (PAT) is widely accepted as a simple memory testing method. The PAT was performed using a “step-through” apparatus (ChengDu Technology & Market Co, LTD) composed of six reaction boxes, allowing for six mice to be tested simultaneously. Here, the latency to enter the dark box for the first time and the latency to enter the dark box after an electric shock was applied to the feet were observed and automatically recorded to probe the learning and memory abilities of the mice. The mice were placed in the illuminated compartment facing away from the dark compartment during the first three days of the training trials. Then, LPS (i.p. 500 μg/kg or 750 μg/kg) was injected 6 h before each daily test during the training phase (7 days). All mice were placed in the illuminated compartment, and upon moving completely into the dark compartment, they received an electric shock (39 V, 3-s duration).

### Climbing pole test

All mice were submitted to the “Pole test” to measure their motor coordination. Briefly, the mice were subjected to five days of training on a pole that was 60 cm in length and 1 cm in diameter with two layers of gauze on the outside. On the sixth day, LPS (i.p. 500 μg/kg or 750 μg/kg) was injected 6 h before the climbing pole test, and the following three times were recorded: the time it took for the mouse to climb down the upper half, the time it took for the mouse to climb down the lower half, and the time it took for the mouse to complete the total length of the pole. If the mouse completed the above three steps within 3 s, 6 s or longer than 6 s, the motor coordination score was 3 points, 2 points, or 1 point, respectively.

### Immunofluorescence staining

To measure the microglia activation and neuronal cell loss in the mouse hippocampus, the mice were perfused with normal saline, followed by 4% paraformaldehyde (PFA) in 0.1 M sodium phosphate buffer (PBS), pH = 7.4. The cerebral tissues were removed, incubated overnight in fixatives, and stored in a 30% sucrose solution. After being transferred to a 30% sucrose solution, the brains were cut into 10-μm-thick sections using a cryostat microtome (Leica CM 1850; Leica Microsystems, Seoul, Korea) and incubated at 37 °C overnight. After three 5-min washes with PBS (pH = 7.4) and permeabilization with Triton X-100 (0.3% in TBST) at room temperature, the brains sections were blocked for 1 h in 1% bovine serum albumin (BSA) solution and overnight at 4 °C with a 1:100 dilution of an antibody against microtubule-associated protein 2 (MAP-2, Millipore Corp, Billerica, MA, USA), 1:200 dilution of an antibody against ionized calcium-binding adapter protein 1 (IBA-1, Millipore Corp, Billerica, MA, USA) and 1:100 dilution of an antibody against amyloid beta 1–42 (Aβ_1–42_, Abcam, Cambridge, UK). On the following day, after the incubation with the primary antibodies, the brain sections were washed three times with PBS (pH = 7.4) for 10 min per wash. Subsequently, the sections were incubated in a 1:200 dilution of a TRITC donkey anti-rabbit secondary antibody and FITC donkey anti-mouse secondary antibody for 1 h at room temperature and then washed with PBS (pH = 7.4) three times for 10 min per wash. Then, the sections were stained with DAPI staining solution for 10 min at room temperature. Finally, fluorescence images were obtained using fluorescence microscopy (Leica Microsystems, Wetzlar, Germany), and the number of cells was analyzed as the number of positive cells/total cells using ImageJ 1.50 software (National Institutes of Health, Bethesda, MD, USA).

### ELISA

The frozen brains were homogenized in 100 mg tissue/mL cold PBS. The samples were centrifuged at 12,000 × g for 15 min. The supernatant was collected for a protein assay using a BCA protein assay reagent kit (PIERCE, Milwaukee, WI). The serum and brain levels of IL-1β (eBioscience, Vienna, Austria), TNF-α (eBioscience Vienna, Austria), PGE_2_ (R&D Systems, Minneapolis, MN), IL-4 (USCN, Wuhan, China), and IL-10 (USCN, Wuhan, China) were measured using ELISA kits according to the manufacturer’s instructions.

### Nitrite (Griess) assay

The frozen brains were homogenized in 100 mg tissue/mL cold PBS. The samples were centrifuged at 12,000 × g for 15 min. The supernatant was collected for a protein assay using a BCA protein assay reagent kit. The levels of NO in the serum and brain were measured as nitrite using the Griess reaction, which was performed in strict accordance with the directions.

### Western blot analysis

The hippocampus tissues were homogenized in RIPA lysis buffer (Bioteke Co, Beijing, China) containing 1 mM phenylmethylsulfonyl fluoride and then centrifuged at 12,000 × *g* for 15 min at 4 °C. The cytoplasmic and nuclear p65 detection were performed according to the NE-PER^®^ instructions (Thermo Scientific, Rockford, IL, USA) to obtain cytoplasmic and nuclear protein. An equal amount of protein was separated via SDS-PAGE and transferred onto a nitrocellulose membrane. The membranes were blocked for 1 h with a 5% skim milk solution and incubated overnight at 4 °C with specific antibodies (1:1000 dilution, Cell Signaling Technology Inc, MA, USA). Then, the membranes were incubated in a 1:15000 dilution of a horseradish peroxidase-conjugated secondary antibody (Cell Signaling Technology Inc, MA, USA) for 1 h at room temperature. The signals were measured with an enhanced chemiluminescence kit (ECL, Millipore, USA) using a gel imaging system (Millipore, Billerica, MA, USA), and the results were visualized using Quantity One software.

### Statistical analysis

Unless otherwise stated, all experiments were performed with triplicate samples and repeated at least three times. The data are presented as the mean ± *SEM* and were analyzed using one-way ANOVA, followed by Tukey’s honest significant difference test. Statistical significance was accepted at *P* < 0.05.

## Results

### General appearance and weight loss

All mice were visually inspected and weighed daily immediately prior to testing. After the LPS administration, the mice exhibited classic signs of sickness behaviors, including decreased locomotion, a hunched posture and anorexia (data not shown).

### LPS-induced cognitive impairment

**Figure 2:**
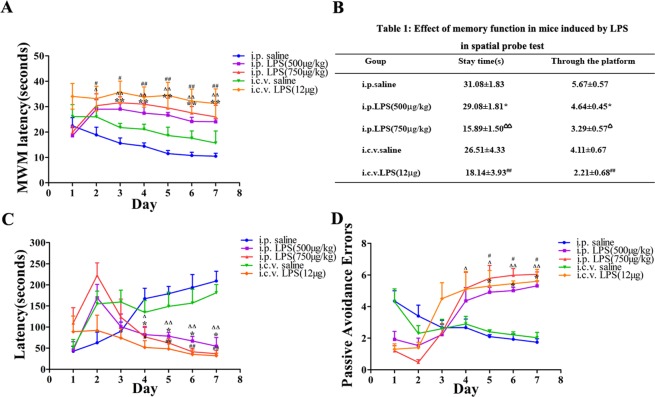
LPS-induced memory defects in the MWM test and passive avoidance performance test. (**A**) Mice showed impaired learning and memory function after injections of LPS during the place-navigation test. (**B**) Mice showed an effect of LPS on memory function during the spatial probe test. C: Latency in the passive avoidance test. D: Error number in the passive avoidance test (n = 10). *P < 0.05, **P < 0.01 LPS-induced (i.p. 500 μg/kg) compared to the i.p. saline group; ^Δ^*P* < 0.05, ^ΔΔ^*P* < 0.01 LPS-induced (i.p. 750 μg/kg) compared to the i.p. saline group; ^#^*P* < 0.05, ^##^*P* < 0.01, LPS-induced (i.c.v. 12 μg) compared to the i.c.v. saline group.

To elucidate the LPS-induced cognitive impairment, i.e., learning and memory, in the mice, a MWM test and PAT were conducted. Over the course of multiple training trials, the mice learned to find the platform, but the LPS-induced mice (24.05 ± 1.12 s, 25.96 ± 4.59 s and 31.22 ± 5.78 s) arrived at the location of the platform more slowly than the control mice (10.47 ± 1.14 s and 15.66 ± 4.70 s), demonstrating that a memory deficiency could be induced by LPS (Fig. 2A). Therefore, during the testing trial (day 2), the mice that had received a single i.p. injection of LPS (500 μg/kg or 750 μg/kg) or i.c.v. LPS (12 μg) spent significantly less time in the quadrant of the platform and on the platform than the mice that received saline (Table 1). Subsequently, in the PAT, we found that the mice given i.p. LPS (500 μg/kg or 750 μg/kg) and the mice given i.c.v. LPS (12 μg) remained in the illuminated compartment for a shorter amount of time (54.64 ± 20.50 s, 37.02 ± 7.55 s and 31.99 ± 6.26 s) than the control mice (209.12 ± 23.11 s and 181.20 ± 19.50 s) (Fig. 2B). In addition, the error number in the PAT was approximately 1.74 ± 0.22 s and 2.03 ± 0.33 s in the control group and 5.31 ± 0.31 s, 6.04 ± 0.15 s and 5.60 ± 0.75 s in the LPS-induced (i.p. 500 μg/kg and 750 μg/kg and i.c.v. 12 μg) mice (Fig. 2C).

**Table 1:** Effect on memory function in mice induced by LPS during a spatial probe test.

Group	Stay time(s)	Times across the platform
i.p. saline	31.08 ± 1.83	5.67 ± 0.57
i.p. LPS (500 μg/kg)	29.08 ± 1.81^*^	4.64 ± 0.45^*^
i.p. LPS (750 μg/kg)	15.89 ± 1.50^ΔΔ^	3.29 ± 0.57^Δ^
i.c.v. saline	26.51 ± 4.33	4.11 ± 0.67
i.c.v. LPS(12 μg)	18.14 ± 3.93^**##**^	2.21 ± 0.68^**##**^

### Motor coordination of LPS-induced mice in the pole test

**Figure 3:**
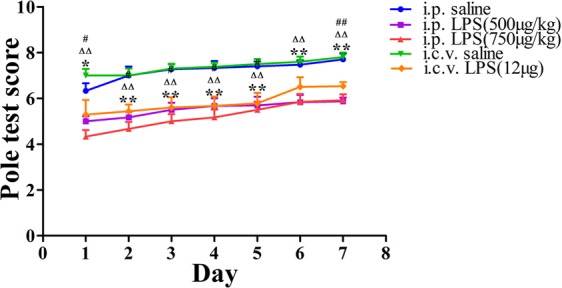
Motor coordination scores in the LPS-induced mouse model as assessed in mice daily. *P < 0.05, **P < 0.01 LPS (i.p. 500 μg/kg) compared to the i.p. saline group; ^Δ^*P* < 0.05, ^ΔΔ^*P* < 0.01 LPS (i.p. 750 μg/kg) compared to the i.p. saline group; ^#^*P* < 0.05, ^##^*P* < 0.01, LPS (i.c.v. 12 μg) compared to the i.c.v. saline group.

The pole test is useful for investigating motor coordination in LPS-induced mice. The mice were subjected to the test daily. As shown in Fig. 3, the motor coordination scores of the LPS-induced mice were significantly decreased following the injection of LPS (*P* < 0.01 compared with those of the control group).

### LPS-induced neuronal cell loss and microglia activation

**Figure 4:**
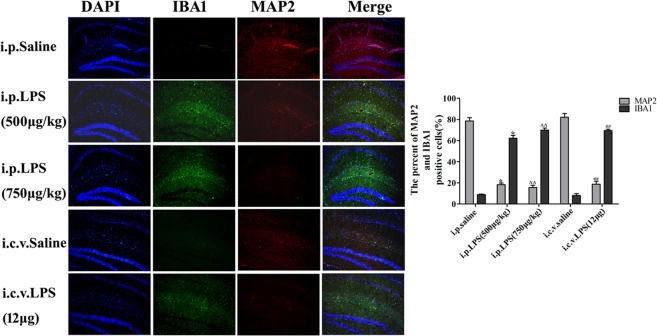
Expression of MAP-2 and IBA-1 in the LPS-induced mouse model of memory and learning impairment. Immunostaining of IBA-1 (*green*) and MAP-2 (*red*) proteins in the hippocampus was performed with specific primary antibodies, quantified images of *n* = 5 per group. Graph is plotted as the mean + *SEM*. *P < 0.05, **P < 0.01 LPS (i.p. 500 μg/kg) compared to the i.p. saline group; ^Δ^*P* < 0.05, ^ΔΔ^*P* < 0.01 LPS (i.p. 750 μg/kg) compared to the i.p. saline group; ^#^*P* < 0.05, ^##^*P* < 0.01, LPS (i.c.v. 12 μg) compared to the i.c.v. saline group.

To verify the relationship between the LPS-induced activation of microglia and neuronal cell loss, we examined the induction of neuronal cell loss by LPS in the hippocampus, which would cause memory deficits. The numbers of IBA1-positive and MAP2-positive cells in the hippocampus (IBA-1 is a specific maker of microglia, and MAP-2 is usually expressed in axons and dendrites of neurons) were used to evaluate microglia activation and neuronal cell loss in LPS-induced mice. We found that LPS (i.p. 500 mg/kg and 750 mg/kg and i.c.v. 12 mg) caused obvious neuronal cell loss labeled by MAP-2 (18.80% ± 1.60%, 15.70% ± 2.10% and 18.80% ± 2.80% *vs* the control group) and microglial activation labeled by IBA-1 (62.2% ± 2.70%, 69.90% ± 1.99% and 69.43% ± 1.42% *vs* the control group; Fig. 4) in the hippocampus.

### Expression of proinflammatory cytokines in LPS-induced mice

**Figure 5:**
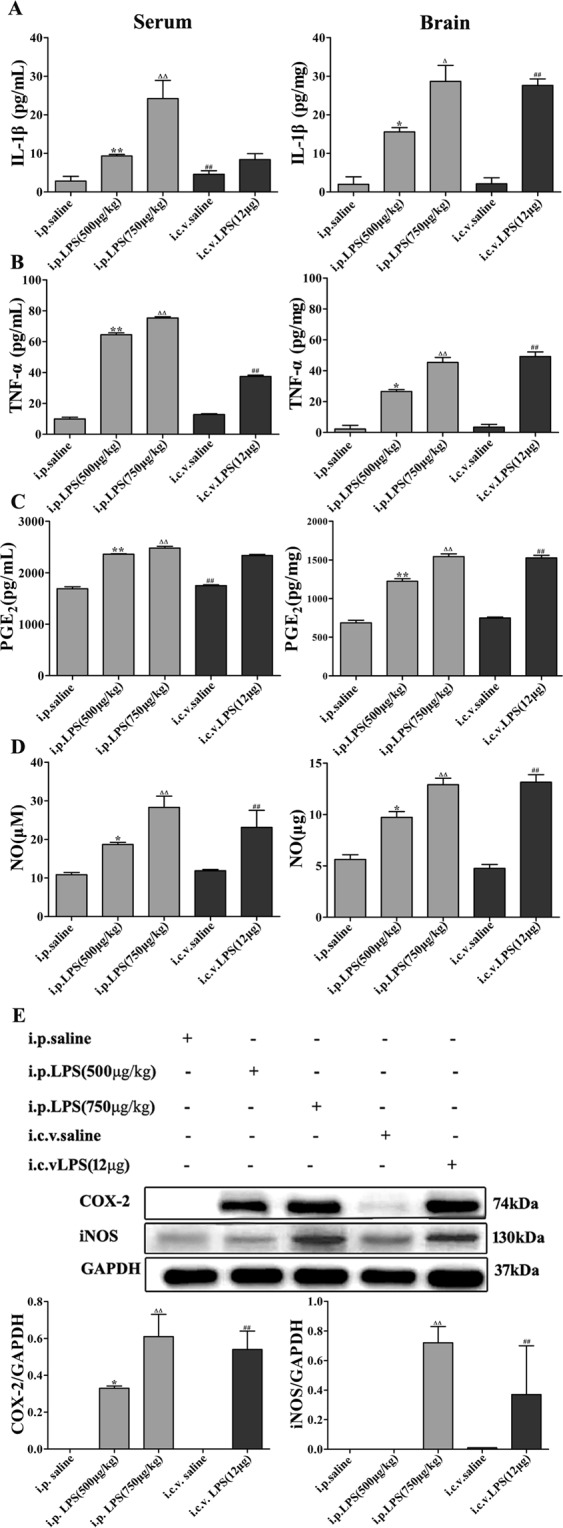
Expression of proinflammatory cytokines in the LPS-induced mouse model of memory and learning impairment. (**A**–**D**) The expression of the proinflammatory cytokines IL-1β, TNF-α, PGE_2_ and NO in serum and brain homogenates. (**E**) LPS-induced expression of COX-2 and iNOS in the brain; the data were determined by Western blotting. The data are described as the mean ± *SEM* (n = 10). *P < 0.05, **P < 0.01 LPS (i.p. 500 μg/kg) compared to the i.p. saline group; ^Δ^*P* < 0.05, ^ΔΔ^*P* < 0.01 LPS (i.p. 750 μg/kg) compared to the i.p. saline group; ^#^*P* < 0.05, ^##^*P* < 0.01, LPS (i.c.v. 12 μg) compared to the i.c.v. saline group.

To investigate the proinflammatory reaction in the LPS-induced mice, we measured the expression levels of proinflammatory cytokines such as TNF-α, IL-1β, PGE_2_ and NO, in the serum and brain homogenates. As shown in Fig. 5A–D, the expression levels of TNF-α and IL-1β in the serum and brain homogenates of the LPS-induced mice were higher than those in the control group, demonstrating that LPS can lead to the occurrence of inflammation and the release of proinflammatory cytokines. Then, we measured the expression levels of PGE_2_ by ELISA and NO by the Griess reaction in LPS-induced mice and observed that the levels of PGE_2_ and NO in the LPS (i.p. 500 μg/kg and 750 μg/kg and i.c.v. 12 μg) groups were higher than those in the controls. Prostaglandins (PGs) are autacoid lipid mediators important for physiological responses and inflammation. Cyclooxygenases (COXs) are key enzymes that catalyze the generation of PGs from arachidonic acid, and there are two isozymes, i.e., COX-1 and COX-2. Furthermore, inducible nitric oxide synthase (iNOS) expression and NO production are thought to participate in the deleterious effects of inflammation^28–30^. To further explore this phenomenon, we measured the protein expression of COX-2 and iNOS using Western blotting. As shown in Fig. 5E, the LPS groups had significantly higher levels of COX-2 and iNOS expression than the controls.

### Expression of anti-inflammatory cytokines in LPS-induced mice

**Figure 6:**
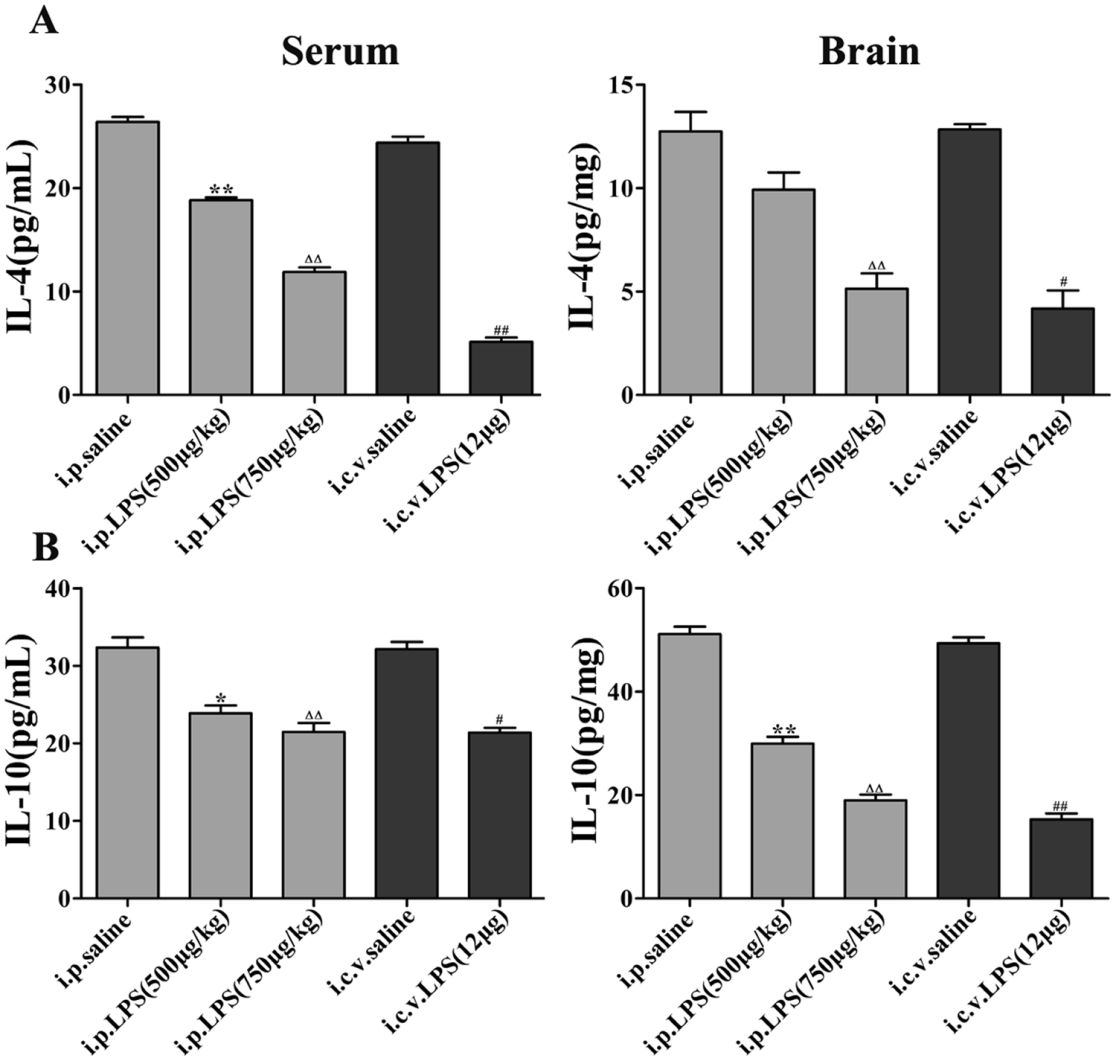
Expression of anti-inflammatory cytokines in the LPS-induced mouse model of memory and learning impairment. The data are described as the mean ± *SEM* (n = 10). *P < 0.05, **P < 0.01 LPS (i.p. 500 μg/kg) compared to the i.p. saline group; ^Δ^*P* < 0.05, ^ΔΔ^*P* < 0.01 LPS (i.p. 750 μg/kg) compared to the i.p. saline group; ^#^*P* < 0.05, ^##^*P* < 0.01, LPS (i.c.v. 12 μg) compared to the i.c.v. saline group.

Subsequently, we investigated the expression of IL-4 and IL-10, which are two important anti-inflammatory cytokines, in the LPS-induced mice. The serum and brain homogenate expression levels of IL-4 and IL-10 in the mice undergoing LPS administration for 1 or 7 consecutive days were significantly decreased compared with the levels in the control group (Fig. 6).

### LPS-induced *NF-κB* signaling pathway activation

**Figure 7:**
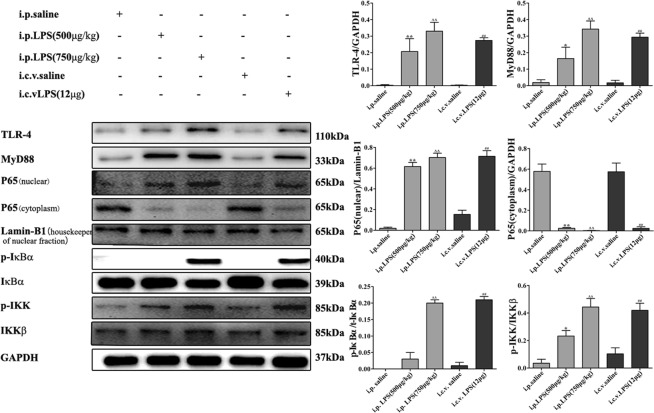
Effects of different treatments on NF-κB-related proteins in brain homogenates. The data are described as the mean ± *SEM* (n = 10). *P < 0.05, **P < 0.01 LPS-induced (i.p.500 μg/kg) compared to the i.p. saline group; ^Δ^*P* < 0.05, ^ΔΔ^*P* < 0.01 LPS-induced (i.p. 750 μg/kg) compared to the i.p. saline group; ^#^*P* < 0.05, ^##^*P* < 0.01, LPS-induced (i.c.v. 12 μg) compared to the i.c.v. saline group.

To characterize the mechanism by which LPS induces the expression of proinflammatory cytokines, the activation of the NF-κB signaling pathway in the brain was determined via Western blotting. The expression of TLR-4 and MyD88 and the phosphorylation of IκBα and IκB kinase (IKK) in the LPS (i.p. 500 μg/kg and 750 μg/kg and i.c.v. 12 μg) groups were significantly higher than those in the control (i.p. saline and i.c.v. saline) groups, and this increase induced the translocation of the NF-κB p65 subunit into the nucleus (Fig. 7). The activation process of the NF-κB signaling pathway includes the phosphorylation of IKK and results in the degradation of IκBα, followed by the subsequent nuclear translocation of the p65 subunit.

### LPS-induced Aβ1–42 generation in mouse brains

**Figure 8:**
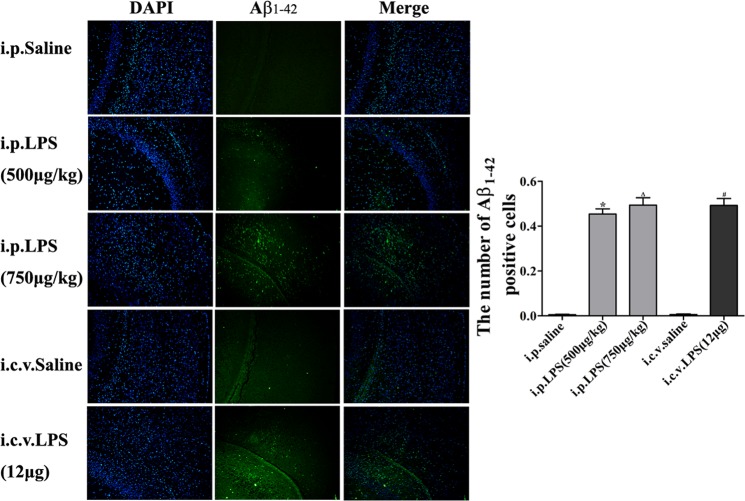
Effect of LPS on Aβ_1–42_ accumulation in the hippocampus. Immunostaining of Aβ_1–42_ (*green*) in the hippocampus was performed with specific primary antibodies, quantified images of *n* = 5 per group. Graph is plotted as the mean + *SEM*. *P < 0.05, **P < 0.01 LPS (i.p. 500 μg/kg) compared to the i.p. saline group; ^Δ^*P* < 0.05, ^ΔΔ^*P* < 0.01 LPS (i.p. 750 μg/kg) compared to the i.p. saline group; ^#^*P* < 0.05, ^##^*P* < 0.01, LPS (i.c.v. 12 μg) compared to the i.c.v. saline group.

We analyzed the Aβ_1–42_ expression in the hippocampus following the LPS injections. The Aβ_1–42_ immunoreactivity observed in the LPS groups was higher than that in the control groups (Fig. 8).

### VIPER attenuates the cognitive impairment, proinflammatory cytokines and NF-κB activity in LPS-induced mice

**Figure 9:**
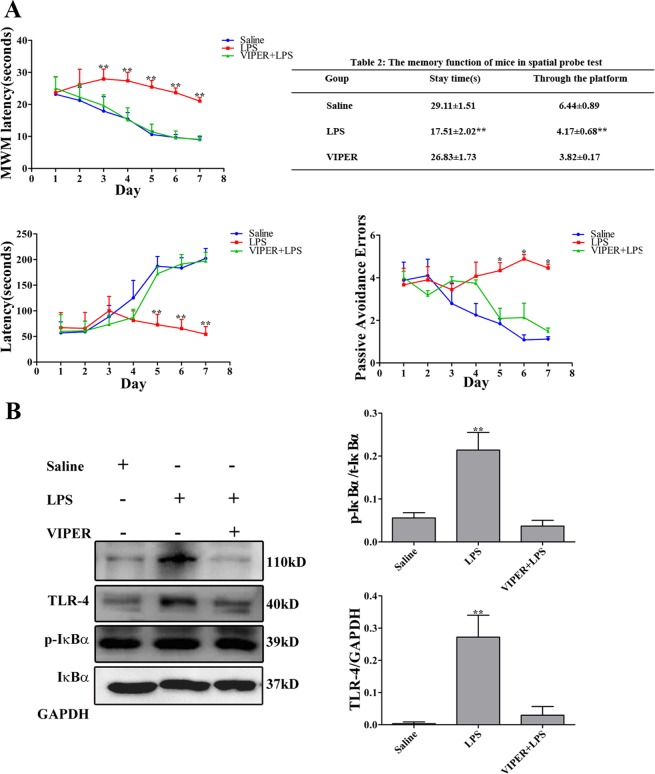
VIPER treatment attenuates cognitive dysfunction and neuroinflammation. (**A**) Effects of VIPER on LPS-induced memory deficit evaluated by the MWM and passive avoidance performance test. (**B**,**C**) VIPER attenuates LPS-induced proinflammatory cytokines and protein accumulation. (n = 10). *P < 0.05, **P < 0.01 LPS-induced (i.p. 750 μg/kg) compared to the i.p. VIPER + LPS group.

Lysakova *et al*.^31^ have identified VIPER, an inhibitor peptide of TLR-4, could directly interact with the TLR-4 adaptor proteins MyD88 adaptor-like (Mal) and TRIF-related adaptor molecule (TRAM). In addition, it has been reported that VIPER abrogated LPS-induced NF-κB activation^32^. Thus, we observed the effect of VIPER on LPS-induced mice. As expected, VIPER-treated mice exhibited a shorter escape latency that the LPS-treated mice. After the final day of the MWM, we performed a probe test to calculate the time spent in the target quadrant zone, thereby testing for the maintenance of memory. The VIPER-treated mice spent more time in the quadrant zone than the LPS-induced mice; the times of passing through the platform were significantly increased. Then, through the passive avoidance test, we tested how long the mice can remember the locations. Notably, the LPS-treated mice showed significantly decreased step-through latency than the VIPER-treated mice. The error number of the VIPER-treated mice was lower than that of the LPS-treated mice. We performed ELISA and Western blotting to detect the proinflammatory cytokines (TNF-α, IL-1β and PGE_2_) and NF-κB signaling pathway-related proteins (TLR-4 and IκBα) in the serum and brain, which consequently indicate the activation of the NF-κB signaling pathway and the occurrence of neuroinflammation. We found that the treatment with LPS elevated the expression of proinflammatory cytokines (TNF-α, IL-1β and PGE_2_) and proteins (TLR-4 and IκBα), but this expression were significantly reduced by the VIPER treatment (Fig. 9).

## Discussion

Neuroinflammation has been suggested to contribute to neurodegenerative diseases and cognitive dysfunction. To date, the exact pathogenesis of many neurodegenerative diseases, such as AD, PD, and MS, are unclear, and effective treatments for these diseases are lacking. Thus, the establishment of an appropriate animal model is very important for researching neuroinflammation-associated cognitive impairment and neurodegenerative diseases. Recently, increasing reports^33^ have shown that the administration of LPS contributes to increased neuroinflammation along with damage to the Blood Brain Barrier (BBB), thereby causing amyloid genesis and memory deficiency. Furthermore, LPS-induced brain inflammation is accompanied by neuronal loss and microglia activation, which induce the release of neurotoxic factors, such as inflammatory cytokines (TNF-α, IL-1β, PGE_2_, etc.). The chronic administration of LPS can cause spatial memory and learning impairment analogous to the cognitive decline observed during AD, which is associated with inflammation and amyloid genesis due to increasing Aβ deposition^31,32,34–38^. Studies^39^ have increasingly demonstrated that LPS-induced models of cognitive impairment, such as AD and PD, are valid for studying the mechanism of cognitive impairment. However, many studies focus on one type of injection method of LPS, a few time points and/or a single dose of LPS. Selecting the appropriate injection method, time point and dose of LPS, is challenging for researchers. Consequently, assessing time- and dose-dependent changes in neuroinflammation and behavior following the administration of LPS is important. Here, we induced neuroinflammation via i.p. or intracerebroventricular (i.c.v.) injections of different doses of LPS to quantify neuroinflammation and behavioral changes and investigated the possible mechanisms of LPS-induced cognitive impairment to provide researchers with more detailed data in this field.

Studies have demonstrated that an LPS-induced mouse model of neuroinflammation is an important tool for deciphering the pathological mechanisms involved in neurodegeneration and testing potential therapeutic molecules. We agree with this perspective. Meanwhile, i.p. and i.c.v. injections of LPS are the most popular methods used to establish animal models. However, there are two drawbacks to the i.c.v. LPS-induced model. First, i.c.v. injections of LPS require a longer trauma recovery period. Second, i.c.v. administration is difficult to perform and is time consuming (Table 2).

**Table 2:** Time required for i.p. and i.c.v. injections of LPS.

	Anesthesia	Operation	Awake
i.p. LPS	—	5 min	—
i.c.v. LPS	10 min	1 μl/5 min	30 min–45 min
		The cannula is left in place for 5 min following injection	

We further performed a comparison with other published LPS injection methods. (1) Yue Hou *et al*.^40,41^ reported that LPS (40 μg) via intrahippocampal injection induced learning and memory impairment and possible changes in microglia and neurons. These authors used the MWM test to detect learning and memory impairment 15 days after intrahippocampal LPS injections. In their two studies, the authors found that the mice in the LPS group took longer to find the platform than did the mice in the control group on the 4th and 5th days, which took longer than our mice (2 days), and the time points of the model were not determined. (2) Hei-Jen Huang *et al*.^42^ reported that intrahippocampal LPS (4 μg) injections induced cognitive dysfunction. Although this model significantly increased the escape latency compared with that observed following intrahippocampal saline injections on the 2nd day of the MWM test, the MWM test was performed on days 22–28 after the intrahippocampal LPS injections. (3) In our studies, the MWM test was conducted six hours after the i.p. or i.c.v. injections of LPS. The mice in the LPS group took longer to find the platform than the mice in the control group on the 2nd day. Meanwhile, intrahippocampal injections are more complicated than intraperitoneal injections and are associated with high difficulty and a low survival rate requiring anesthetization. Furthermore, the model takes a shorter time to succeed than those in the other studies.

The MWM was described 20 years ago as a method of investigating spatial learning and memory in laboratory animals^43,44^ and has become one of the most popular research tools in various subfields of behavioral neuroscience, especially in the AD research area. Both the i.p. and i.c.v. administration of LPS result in memory loss. Subsequently, we examined the passive avoidance performance of the mice. The amount of time the mice remained in the illuminated compartment was indicative of their memory ability. Here, the i.p. and i.c.v. LPS-treated mice stayed in the light compartment for a shorter time than the i.p. and i.c.v. saline-treated mice. Additionally, we found that as the dose increased, the mice showed LPS dose-dependent memory deficiency. These results indicate that the chronic neuroinflammation model established by systemic LPS injections in this study could induce cognitive impairment.

Neurodegenerative patients are known to exhibit a lower expression of acetylcholinesterase neurons and choline acetyltransferase (ChAT) in the hippocampus and acetylcholine (ACh) synthesis, release and uptake dysfunction. Therefore, we used the pole test to observe the motor coordination of the mice. We found that the i.p. LPS and i.c.v. LPS groups received a lower score than the control groups in the pole test.

To further identify the causes of the behavioral changes in the mice, we investigated the neurons and microglia in the hippocampus of the LPS-induced mice using immunofluorescence staining. IBA-1, which has been used to detect microglia in normal or pathological lesions in mice^45,46^, has also been found to play a proinflammatory role^47,48^. MAP-2 is typically expressed in the axons and dendrites of neurons^49^ and is critical for maintaining the spacing between microtubules^50,51^. Furthermore, MAP-2 immunoreactivity has been reported to be a credible and quantifiable marker of early neuronal injury. In our experiment, 7 days after the LPS injections, we found that the number of microglia (IBA1-positive cells) was higher and the number of neurons (MAP2-positive cells) in the LPS-induced mice were lower than those in the control groups.

The activation of serum and brain homogenate cytokine signaling in response to the peripheral or central administration of LPS has been repeatedly demonstrated to mediate LPS-induced sickness behavior^52–54^. In the present study, the i.p. and i.c.v. injections of LPS induced sickness behavior as measured by body weight loss and decreased social exploration, and this effect was associated with the increased expression of the steady-state transcripts of TNF-α, IL-1β, NO and PGE_2_ in the serum and brain homogenates. We found that the expression of proinflammatory cytokines (TNF-α and IL-1β) in the LPS-induced mice was higher and the expression of anti-inflammatory cytokines (IL-4 and IL-10) was lower than those in the control mice. In addition, LPS, Aβ, IFNγ, etc. induce microglia synthesis of NO. Therefore, we further showed that iNOS and NO expression were increased by using Western blotting and the Griess assay, respectively. Furthermore, COXs are the rate-limiting enzymes in the synthesis of PGs and thromboxanes. Two isoforms of COX, i.e., COX-1 and COX-2, have been previously described, and the expression of COX-2 is upregulated by a wide variety of stimuli, such as inflammatory mediators.

Toll-like-receptor 4 (TLR-4), which is a lipopolysaccharide (LPS) receptor, is an important molecule that mediates several inflammatory pathways^55^. The activation of TLR-4 leads to NF-κB activation, which is associated with the production of proinflammatory cytokines through a Myeloid differentiation factor 88 (MyD88)-dependent pathway^56^. To characterize the mechanism by which LPS induces proinflammatory cytokine expression, we detected the expression of NF-κB signaling pathway-related proteins in the brain via Western blotting. As shown in Fig. 7, IκBα and IKK phosphorylation in the LPS groups was significantly higher than that in the control groups, and the translocation of the NF-κB p65 subunit into the nucleus was induced. The transcription factor p65 is involved in the transcriptional regulation of these proinflammatory mediators. Under physiological conditions, p65 is retained in the cytoplasm by binding to IκBα. Upon LPS stimulation, the activation of the NF-κB signaling pathway occurs through the phosphorylation of IκBα, resulting in the nuclear translocation of p65.

Aβ, which is the most important component of neuritic plaques in AD, appears to be the primary mediator of the neuroinflammation characterizing the disease^57–59^. AD is known to be associated with the accumulation of Aβ_1–42_ before tau pathology and clinical symptoms become apparent^60^. Here, we found that LPS-treated brains showed high levels of Aβ_1–42_, suggesting that this animal model of systemic inflammation induced by LPS may be useful for researching the pathogenesis of AD. More interestingly, these findings suggest that another mechanism of neuroinflammation by LPS may be partially due to the production of Aβ by activated microglia. Subsequently, Aβ stimulates microglia, which can result in neuroinflammation. Some studies have indicated that the brains of mutant presenilin 2 mice (a genetic AD model) showed increased inflammation and Aβ accumulation accompanied by neuronal cell death, and we also demonstrated this phenomenon in our mouse model. Nevertheless, the mechanism by which LPS induces Aβ accumulation and the relationship between microglia and Aβ are unclear and require further investigation.

Based on the results of our study, LPS induces neuroinflammation via the TLR-4 signaling pathway, leading to cognitive impairment. To prove the causal relationship between neuroinflammation and neurodegeneration, we further used a TLR-4 inhibitor to inhibit NF-κB pathway signaling activation. As expected, the specific TLR-4 inhibitor VIPER remarkably attenuated the LPS-induced neuroinflammation; moreover, VIPER abolished the cognitive impairment following neuroinflammation.

In conclusion, our present study showed that LPS injections stimulate microglia through the activation of the NF-κB signaling pathway. Systemic inflammation and neuroinflammation due to LPS injections cause an elevation in Aβ levels and neuronal cell death, finally resulting in cognitive impairment. Therefore, these results support the hypothesis that systemic inflammation is involved in the progression of cognitive impairment, such as AD and PD, and anti-inflammatory treatment might be useful for its prevention.

## References

[1] Allison DJ, Ditor DS (2014). The common inflammatory etiology of depression andcognitive impairment: a therapeutic target. J Neuroinflammation..

[2] Nguyen MD, Julien JP, Rivest S (2002). Innate immunity: The missing link in neuroprotection and neurodegeneration?. Nat Rev Neurosci..

[3] McGeer PL (1988). Reactive microglia are positive for HLA-DR in the substantia nigra of Parkinson’s and Alzheimer’s disease brains. Neurolog..

[4] Kiefer R (1995). Transforminggrowth factor-beta 1: a lesion-associated cytokine of the nervous system. Int J Dev Neurosci..

[5] Imai F (2007). Neuroprotective effect of exogenous microglia in global brain ischemia. J Cereb Blood Flow Metab.

[6] Lambertsen KL (2009). Microglia protect neurons against ischemia by synthesis of tumor necrosis factor. J Neurosci..

[7] Gavilán MP (2007). Molecular and cellular characterization of the age-related neuroinflammatory processes occurring in normal rat hippocampus: potential relation with the loss of somatostatin GABAergic neurons. J Neurochem..

[8] Jiménez S (2008). Inflammatory response in the hippocampus of PS1M146L/APP751sl mouse model of Alzheimer’s disease: age-dependent switch in the microglial phenotype from alternative to classic. J Neurosci..

[9] Crain J, Nikodemova M, Watters JJ (2013). Microglia express distinct M1 and M2 phenotypic markers in the postnatal and adult central nervous system in male and female mice. J Neurosci Res..

[10] Hirsch EC, Hunot S, Damier P (1988). Glial cells and inflammation in Parkinson’s disease: a role in neurodegeneration?. Ann Neurol..

[11] Nagatsu T, Mogi M, Ichinose H (2000). Cytokines in Parkinson’s disease. J Neural Transm Suppl..

[12] Liu B, Hong JS (2003). Role of microglia in inflammation-mediated neurodegenerative diseases: mechanisms and strategies for therapeutic intervention. J Pharmacol Exp Ther..

[13] Li X, Buxbaum JN (2011). Transthyretin and the brain re-visited: is neuronal synthesis of transthyretin protective in Alzheimer’s disease?. Mol Neurodegener..

[14] Eikelenboom P, van Gool WA (2004). Neuroinflammatory perspectives on the two faces of Alzheimer’s disease. J Neural Transm..

[15] Koning N (2007). Downregulation of macrophage inhibitory molecules in multiple sclerosis lesions. Ann Neurol..

[16] Krause DL, Müller N (2010). Neuroinflammation, microglia and implications for antiinflammatory treatment in Alzheimer’s disease. Int J Alzheimers Dis..

[17] Beutler B (2000). TLR4: central component of the sole mammalian LPS sensor. Curr Opin Immunol..

[18] Lehnardt S (2002). The toll-like receptor TLR4 is necessary for lipopolysaccharide-induced oligodendrocyte injury in the CNS. J Neurosci..

[19] McGeer PL, McGeer EG, Yasojima K (2000). Alzheimer disease and neuroinflammation. J Neural Transm Suppl..

[20] Mrak RE, Griffin WS (2005). Glia and their cytokines in progression of neurodegeneration. Neurobiol Aging..

[21] Lysakova-Devine T (2010). Viral inhibitory peptide of TLR4, a peptide derived from vaccinia protein A46, specifically inhibits TLR4 by directly targeting MyD88 adaptor-like and TRIF-related adaptor molecule. J Immunol.

[22] Choi DY (2012). Obovatol attenuates LPS-induced memory impairments in mice via inhibition of NF-κB signaling pathway. Neurochem Int..

[23] Shaw KN, Commins S, O’Mara SM (2001). Lipopolysaccharide causes deficits in spatial learning in the water maze but notin BDNF expression in the rat dentate gyrus. Behav Brain Res..

[24] Arai K (2001). Deterioration of spatial learning performances in lipopolysaccharide-treated mice. Jpn J Pharmacol..

[25] Oitzl MS (1993). Interleukin-1 beta, but not interleukin-6, impairs spatial navigation learning. Brain Res..

[26] Haley TJ, McCormick WG (1957). Pharmacological effects produced by intracerebral injection of drugs in the conscious mouse. British Journal of Pharmacology and Chemotherapy..

[27] Morris R (1984). Developments of a water-maze procedure for studying spatial learning in the rat. J Neurosci Methods..

[28] Gross SS, Wolin MS (1995). Nitric oxide: pathophysiological mechanisms. Ann Rev Physiol..

[29] Smith WL, Marnett LJ (1991). Prostaglandin endoperoxidesynthase: structure and catalysis. Biochim Biophys Acta..

[30] Seibert K (1995). Mediation of inflammation by cyclooxygenase-2. Agents and Actions Suppl..

[31] Wood AJJMD, Cummings JLMD (2004). Alzheimer’s disease. N Engl J Med.

[32] Glass CK (2010). Mechanisms underlying inflammation in neurodegeneration. Cell..

[33] Noh H, Jeon J, Seo H (2014). Systemic injection of LPS induces region-specific neuroinflammation and mitochondrial dysfunction in normal mouse brain. Neurochem Int..

[34] Lee YB, Nagai A, Kim SU (2002). Cytokines, chemokines,and cytokine receptors in human microglia. J Neurosci Res..

[35] Linnartz B, Wang Y, Neumann H (2010). Microglial immunoreceptor tyrosine-based activation and inhibition motif signaling in neuroinflammation. Int Alzheimer Dis..

[36] Neuroinflammation Working Group (2000). Inflammation and Alzheimer’s disease. Neurobiol Agin..

[37] Eikelenboom P (2002). Neuroinflammation in Alzheimer’s disease and prion disease. Glia..

[38] Qin L (2007). Systemic LPS causes chronic neuroinflammation and progressive neurodegeneration. Gli..

[39] McGeer EG, McGeer PL (2003). Inflammatory processes in Alzheimer’s disease. Prog Neuropsychopharmacol Biol Psychiatr..

[40] Hou Y (2016). Minocycline protects against lipopolysaccharide-induced cognitive impairment in mice. Psychopharmacology (Berl).

[41] Hou Y (2014). Pterostilbene attenuates lipopolysaccharide-induced learning and memory impairment possibly via inhibiting microglia activation and protecting neuronal injury in mice. Prog Neuropsychopharmacol Biol Psychiatry..

[42] Huang, H. J. *et al*. Exendin-4 protected against cognitive dysfunction in hyperglycemic mice receiving an intrahippocampal lipopolysaccharide injection. *PLoS One*.), e39656, 10.1371/journal.pone.0039656(2012). 10.1371/journal.pone.0039656 PMC3402484 22844396

[43] D’Hooge R, De Deyn PP (2001). Applications of the Morris water maze in the study of learning and memory. Brain Research Reviews..

[44] Morris RGM (1981). Spatial localization does not require the presence of local cues. Learn & Motiv..

[45] Ito D (1998). Microglia-specific localisation of a novel calcium binding protein, Iba1. Mol Brain Res..

[46] Ito D (2001). Enhanced expression of Iba1, ionized calcium-binding adapter molecule 1, after transient focal cerebral ischemia in rat brain. Stroke..

[47] Yang ZF (2005). Allograft inflammatory factor-1 (AIF-1) is crucial for the survival and pro-inflammatory activity of macrophages. Int Immunol..

[48] Tian Y, Kelemen SE, Autieri MV (2006). Inhibition of AIF-1 expression by constitutive siRNA expression reduces macrophage migration, proliferation, and signal transduction initiated by atherogenic stimuli. Am. J. Physiol. Cell Physiol..

[49] Gabriel R, Wilhelm M, Straznicky C (1992). Microtubule-associated protein 2 (MAP2)-immunoreactive neurons in the retina of Bufo marinus: colocalisation with tyrosine hydroxylase and serotonin in amacrine cells. Cell Tissue Res..

[50] Harada A (2002). MAP2 is required for dendrite elongation, PKA anchoring in dendrites, and proper PKA signal transduction. J Cell Biol..

[51] Teng J (2001). Synergistic effects of MAP2 and MAP1B knockout in neuronal migration, dendritic outgrowth, and microtubule organization. J Cell Biol..

[52] Dantzer R (2008). From inflammation to sickness and depression: when the immune system subjugates the brain. Nature Reviews Neuroscience..

[53] Kelley K (2003). Broussard SR: Cytokine-induced sickness behavior. Brain Behavior and Immunity..

[54] Dantzer R, Kelley K (2007). Twenty years of research on cytokine-induced sickness behavior. Brain Behavior and Immunity..

[55] Nair A (2014). Role of TLR4 in lipopolysaccharide-induced acute kidney injury: Protection by blueberry. Free Radic Biol Med..

[56] Yamamoto M (2003). TRAM is specifically involved in the Toll-like receptor 4-mediated MyD88-independent signaling pathway. Nat Immunol..

[57] Lynch MA (2014). The impact of neuroimmune changes on development of amyloid pathology; relevance to Alzheimer’s disease. Immunology..

[58] Akiyama H (2000). Inflammation and Alzheimer’s disease. Neurobiol. Aging..

[59] Wyss-Coray T (2006). Inflammation in Alzheimer disease: driving force, bystander or beneficial response?. Nat Med..

[60] Eikelenboom P (2011). The Early involvement of the innate immunity in the pathogenesis of Alzheimer’s disease: Neuropathological, epidemiological and genetic evidence. Curr Alzheimer Res..

